# Development of mental health first aid guidelines for problem drinking: a Delphi expert consensus study in Argentina and Chile

**DOI:** 10.1186/s12888-022-03749-x

**Published:** 2022-02-12

**Authors:** Martín Agrest, Thamara Tapia-Muñoz, Esteban Encina, Judith Wright, Sara Ardila-Gómez, Rubén Alvarado, Eduardo A. Leiderman, Nicola Reavley

**Affiliations:** 1 Proyecto Suma, Güemes, 4130 (1425) Ciudad Autónoma de Buenos Aires, Argentina; 2 Department of Behavioural Science and Health, University College London, London, UK; 3 Escuela de Salud Pública, Facultad de Medicina, Universidad de Chile, Santiago, Chile; 4 Melbourne School of Population and Global Health, Centre for Mental Health, University of Melbourne, Victoria, Australia; 5 Instituto de Investigaciones, Facultad de Psicología, Universidad de Buenos Aires, Buenos Aires, Argentina; 6 Departamento de Neurociencias, Facultad de Ciencias Sociales, Universidad de Palermo, Buenos Aires, Argentina

**Keywords:** Alcohol use disorders, Mental Health First Aid (MHFA), Cultural adaptation, Delphi study, Chile, Argentina

## Abstract

**Background:**

Among all psychoactive substances, alcohol consumption presents the most significant public health problem and is a leading risk factor for overall disease burden in Latin America. However, most people who meet criteria for a substance use disorder do not receive treatment in primary or secondary care sources. Community members can play a role in helping people to seek help as they are likely to encounter people experiencing problem drinking and recognize the signs. However, many do not have adequate mental health first aid knowledge or skills to provide help. We aimed to culturally adapt the existing English-language mental health first aid guidelines for helping someone with problem drinking for Argentina and Chile.

**Methods:**

The Delphi consensus method was used to determine the importance of helping actions translated from the English-language guidelines and to add new actions suggested by expert panellists. The importance of each statement was rated by two expert panels. Panel one included people with lived experience (either their own or as a support person, n = 23) recruited in Argentina and panel two included health professionals (n = 31) recruited in Argentina and Chile.

**Results:**

Overall, 165 helping actions were endorsed by panellists across two consecutive survey rounds. Endorsed items included 132 of the 182 items translated into Spanish from the English-language guidelines and 33 of the 61 new items generated from panellists’ comments in the first survey round.

**Conclusions:**

While there were some similarities in recommended helping actions between English-speaking countries, and Argentina and Chile, key differences were seen in attitudes to low-risk drinking. While there was a relatively high level of agreement between health professionals and people with lived experience, some divergence of opinion was seen, particularly in the area of commitment to recovery as a condition for help. Future research should explore the implementation of the guidelines.

**Supplementary Information:**

The online version contains supplementary material available at 10.1186/s12888-022-03749-x.

## Background

Among all psychoactive substances, alcohol consumption presents the most significant public health problem and is a leading risk factor for overall disease burden in Latin America [1] According to the World Health Organization (WHO), alcohol consumption levels in the Americas Region are 40% higher than the global average, while abstention rates for both men and women are consistently lower. Moreover, alcohol is the leading risk factor for death and disability among people aged 15–49 [2]. Alcohol has many health-related effects since it increases risk of infectious diseases, major non-communicable diseases including cancer [3] and coronary artery disease [4], liver cirrhosis [5], mental health problems including suicide [6] and external causes of harm including injuries, violence, and homicide [7, 8]. Beyond the individual disease burden, alcohol-related harm adversely affects households, family and friends, and wider community settings including workplaces [9]. While epidemiological data on alcohol consumption in Latin America are relatively scarce, it has been estimated that alcohol consumption causes over 10% of the disability-adjusted life years (DALYs) in the region [10]. In 2020, the Americas had the second-highest percentage of DALYS attributable to alcohol consumption when compared to the other WHO Regions and the highest rates of alcohol-attributable deaths per 100,000 due to alcohol use disorders [11].

Argentina and Chile rank among the countries in South America with the highest percentage of heavy episodic drinking in youths between 15 and 19 years old [2]. In Chile, alcohol use disorder makes a greater contribution to death and disability than other mental disorders, accounting for 3.3% of total healthy Years of Life Lost (YLL) in the country [12]. The most recent epidemiological study showed the 12-month prevalence rate of alcohol misuse among adults to be 1.9% (0.3% with dependency), with rates of 3.0% in men and 0.8% in women [13]. In Argentina, the latest available information showed that 3.6% of all deaths in the population were attributed to alcohol consumption [14], while the latest epidemiological study showed that alcohol use (with or without dependency) had a 12-month prevalence rate of 1.5% (0.3% with dependency) [15, 16]. The 2018 National Survey of Risk Factors showed that, between 2009 and 2018 heavy episodic drinking rose from 8.9% to 13.3% [17].

There is also some evidence that the COVID-19 pandemic, which hit the region later and harder than other regions may have impacted rates of alcohol use [18]. While a PAHO) study indicated that alcohol use was lower in the Americas during the pandemic [11], other studies suggest that it may have increased in Argentina [19–21], with 40% to 45% of the population (depending on the study), declaring that they had increased their drinking. A recent study among health care workers in Chile showed that 34.5% had increased alcohol use while 38.1% consumed alcohol at the same level [22].

Addressing the burden of disease related to alcohol use in Chile and Argentina is likely to require a multi-faceted approach incorporating environmental strategies to reduce alcohol access and use as well as those that promote easier access to specialty care and treatment, both through adequate provision of services as well as strategies to promote help seeking among the public [23].

### Current policies and community-based support strategies in Chile and Argentina

Current policies in Chile and Argentina support the integrated treatment of mental health and substance use problems. In the Chilean National Health Fund (FONASA)-funded health system, alcohol problems are typically managed in the regional primary care system, with those needing inpatient treatment accessing this through specialized rehabilitation centres, which support treatment of people with mental health problems, substance use problems and co-occurring problems. In Argentina, alcohol misuse is considered to be one of several “problematic consumptions” (“consumo problemático”) and falls under both the jurisdiction of the Minister of Mental Health and Substance Abuse and the National Argentinian Secretary of Integral Policies on Drugs (SEDRONAR) [24]. While public mental health care is generally decentralized and treatment is provided at provincial or municipal levels, federal hospitals are involved in the provision of free-of-charge speciality care for people with alcohol problems. The national strategy is aligned with National Guidelines for Health Policies in the Prevention and Fight against Excessive Alcohol Consumption [25] under Act 1170/2010. This strategy has not been able to develop successful early intervention strategies and reverse the rising trend of alcohol consumption.

Community-based approaches to tackling alcohol problems differ between Chile and Argentina. Between 1967 and 1973, Chile had an Intracommunity Psychiatry Program in Santiago, which involved health professional training of community leaders who in turn, trained people with lived experience of problem drinking. Nowadays, programs are mostly delivered by health professionals [26, 27]. In Argentina, the Institutional Alcoholism Groups (Grupos Institucionales de Alcoholismo [GIA]) has been in operation since the 1980s. This abstinence-based approach considers the biological, psychological, and sociological dimensions of a person’s alcohol use. It involves the bringing together of a therapeutic community of trained lay people, family members and other community resources. The aim of the GIA is to help the person not just to stop drinking alcohol but to reformulate their life into an active and productive life one that does not involve drinking alcohol [28]. Alcoholics Anonymous (AA), which is globally recognized for the efficacy of its “twelve steps”, also has a widespread presence in Argentina with groups in every province and region. AA groups have been considered crucial for recovery from problem drinking despite resistance from mental health professionals, who have concerns about the lack of attention to the causes of alcoholism and that AA may discourage treatment from mental health professionals. In Chile, while there are “Alcoholic rehabilitation groups” with a similar orientation to AA, they are not anonymous, and they integrate the immediate community (neighbourhood) and the person’s relatives. These groups are used mostly by higher-income people because they are private services and are not connected to the public health network.

### Help seeking for alcohol problems

Even in settings where effective and evidence-based treatments are available, many people with substance use problems are never diagnosed or do not access timely and/or appropriate treatment. Alcohol use disorders are among the mental disorders with the lowest treatment rates worldwide [29]. In Latin America, compared to other mental disorders, substance use disorders have the largest treatment gap, with studies finding 83.7% to 91% of people who meet the criteria for a disorder not receiving treatment from primary or secondary care sources [30, 31]. Recent data suggest that the Chilean National Health Fund (FONASA)-funded health system only covers approximately 10% of the total population in need of treatment, though treatment retention rates (defined as those who remain in treatment for at least one year) have improved the past few years [32]. The last psychiatric epidemiological study in Argentina [15] showed that the use of mental health and substance misuse services by individuals with alcohol misuse with dependence was as low as 14.5%.

However, research exploring reasons for low treatment-seeking for alcohol use problems in the Argentinian and Chilean contexts is limited. Local studies examining individual barriers to care for mental health problems more broadly have identified poor mental health literacy (e.g., beliefs that the problem will resolve itself and that treatment is not helpful), stigma, financial burden, and lack of trust in the health care system [30, 33–35].

### Mental Health First Aid

Evidence-based interventions that improve mental health literacy, reduce stigma and educate people in how to provide support to a person at risk of developing a mental illness or substance use problem may play a role in improving rates of service use and thereby, reducing the burden of disease related to problem drinking [36]. Because of the high prevalence of mental health problems, including alcohol problems, community members are likely to come into contact with someone who is developing a problem and may play a role in assisting the person to seek professional help. In recognition of this, and of the fact that many people may lack the appropriate skills to help, Kitchener and Jorm [37, 38] developed the Mental Health First Aid (MHFA) training courses to educate people about an appropriate response to someone developing a mental health problem or in a mental health crisis (such as suicide). Similar, to other health problems or conditions, mental health first aid for problem drinking is defined as the help provided by a member of the community to someone who may be developing, or may already have, a drinking problem, or is in an alcohol related crisis (e.g., alcohol poisoning) [39]. This aid is provided until professional help is available, or until the crisis is resolved. It should be noted that evidence from meta-analyses has shown that MHFA training is effective in improving mental health literacy, reducing stigma and promoting help-seeking [40]. The content of the MHFA training course has been informed by the use of Delphi expert consensus studies with health professionals and people with lived experience (either their own or as carers). Delphi expert consensus studies enable the gathering of practice-based evidence and are useful in cases where randomised controlled evidence is unavailable or infeasible to collect [41]. Moreover, they allow for the assessment of agreement between groups whose views might be expected to differ in some areas. They also allow each participant an equal voice in the process and thus do not prioritise the views of one person or group over another, which is particularly important in capturing the views of people with lived experience of mental health or substance use problems. This method has been successfully used to culturally adapt guidelines for problem drinking in China [42] and Brazil [43],

MHFA training has been widely disseminated in high-income Western countries with relatively well-resourced health systems, although less is known about its appropriateness for use in low and middle-income countries [44]. In such settings, MHFA training may be of value as one of a set of initiatives based on utilizing non-traditional workers to provide, or assist in the provision of, mental health services [45]. In addition to other benefits (e.g., tackling poor mental health literacy and stigma) these initiatives may address the limited number of mental health care providers in many regions and can potentially assist in decreasing their workload. Such interventions must be culturally appropriate, taking into account the culture and health systems of the countries in which they might be implemented.

Thus, the aim of this study was to use the Delphi expert consensus methodology to culturally adapt guidelines for lay members of the community interested in providing first aid to someone with alcohol use problems in Chile and Argentina.

## Methods

**Fig. 1:**
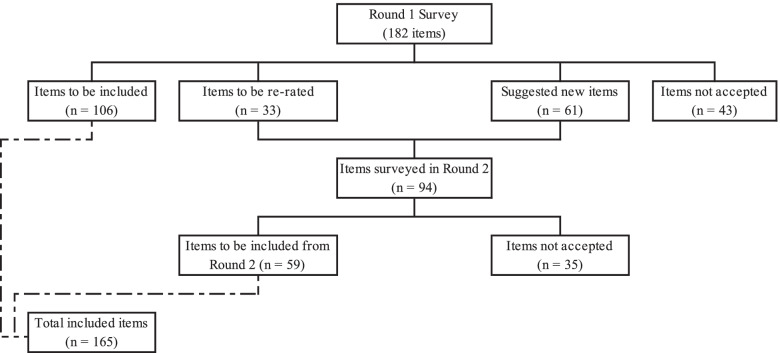
Number of statements included, re-rated and excluded in the two survey rounds

This Delphi study was conducted in four stages: (1) Round 1 survey development; (2) Expert panel member recruitment; (3) Data collection and analyses for the round 1 and 2 surveys; and (4) Guideline’s development. The numbers of statements included, re-rated and excluded in the two survey rounds are shown in Fig. 1.

### Round 1 survey development

The questionnaire for Round 1 was developed by translating statements that were endorsed for inclusion in the mental health first aid guidelines used in English-speaking countries for assisting a person with problem drinking [39]. All 182 items from these guidelines were translated into Spanish and edited by bilingual mental health professionals from Australia, Chile, and Argentina to ensure they were appropriately adapted for the Argentinian and Chilean contexts.

The Round 1 survey consisted of five main sections: (1) Problems with alcohol (20 items), which included items on recognizing patterns of alcohol use (e.g., problem drinking, high-risk drinking, alcohol misuse and dependence) and understanding factors related to approaching the person, and managing the person's unwillingness to change their drinking patterns or seek professional help; (2) Talking to the person about their drinking (39 items), which included items on providing practical tips on approaching the person and managing unwillingness to change in close family or friends; (3) Professional help (49 items), which included items on signs indicating that the person needs professional help, facilitating professional help seeking, and dealing with social pressure to drink; (4) The intoxicated person (67 items), which included items on recognising and understanding alcohol intoxication, practical tips for helping, talking and getting the person home, managing aggression, and managing alcohol-related medical emergencies; and (5) Alcohol withdrawal (7 items), which included items on recognizing and understanding alcohol withdrawal, and seeking medical help.

### Expert panel member recruitment

People with lived experience, either their own or as support people, and health professionals with expertise or experience in problem drinking were recruited by four members of the research team (MA and EL, Argentina; TT and EE, Chile). People with lived experience (Panel one) were only recruited in Argentina due to logistic considerations related to a larger Mental Health First Aid study in these countries, while health professionals (Panel two) were recruited in both Chile and Argentina. Lived experience experts included former GIA participants (with experience helping other people or with experience receiving help through those groups) and AA participants. Both panels were invited to participate using a literal translation of the Australian invitation letter asking to provide their opinions on the actions relating to how to help someone who is developing a mental health problem or who is in a mental health crisis (“brindar sus opiniones sobre las acciones relacionadas con la forma de ayudar a alguien que está desarrollando un problema de salud mental o que se encuentra en una crisis de salud mental”). A broad definition of “person who experience problem drinking” (“persona que puede estar experimentando problemas con el consume de alcohol”) was adopted in the invitation with no additional clarifications. Panelists were recruited through snowballing. Members of the research team distributed information about the study to their personal contacts, who were then encouraged to pass it to others with appropriate experience. Participants who met the following criteria were eligible to participate in an expert panel:

- Aged 18 years old and above.
- Lived experience expert panel—self-identified as having an experience of problem drinking or caring for a person with problem drinking.
- Health professional expert panel—have more than four years of experiencing working as a health care professional with knowledge in problem drinking. Types of eligible professions included, but were not limited to – general practitioners, nurses, psychiatrists, psychologists, or social psychologists.

As both survey rounds were done during the Covid-19 pandemic, participants provided informed consent via email or WhatsApp (an American freeware platform for instant messaging widely used in mobile phones in Chile and Argentina) and included a picture of their signature along with that of a witness on the informed consent form.

### Data collection and analysis

Data were collected in two consecutive survey rounds, with the first round taking place between March 15, 2020, and August 24, 2020, and the second round between September 25, 2020, and January 6, 2021. The Round 1 survey was administered in person via paper-based copies or online via Qualtrics. Due to the COVID-19 pandemic, the Round 2 surveys were administered online only.

Experts rated each item on a 5-point Likert scale (1 = essential, 2 = important, 3 = unsure, 4 = unimportant, 5 = should not be included) according to how important they believed it was for each statement to be included in the mental health first aid guidelines for problem drinking in the Argentinian and Chilean contexts. Items were accepted for inclusion in the final guidelines if at least 80% of participants from both panels rated them as “essential” or “important”. Items were re-rated in the second round of the survey if they were rated as “essential” or “important” by 70.0 – 79.9% of panellists from at least one expert panel in the Round 1 survey. Items were excluded if they were rated as “essential” or “important” by less than 70% of participants from at least one panel.

In the Round 1 survey, open text response boxes were displayed after every 10 items or at the end of each sub-section in the surveys for comments or suggestions for new items that participants thought would be important to add to the final guidelines. New items were also generated by MA and TT from the suggestions in Round 1. These newly generated items were discussed in further detail with NR before being included for rating in Round 2 to ensure that they were new ideas and were clear and actionable. Items that had not received at least 80% endorsement and for which expert panellists made suggestions relating to language or further clarification were re-phrased and included in Round 2 for re-rating. After each survey round, participants were sent a summary of the results. These included endorsement ratings for each item by expert panel group.

### Guidelines development for Chile and Argentina

MA and TT wrote endorsed statements from the two survey rounds into a guidelines document. The other co-authors suggested changes in their native tongue before a final Spanish guidelines draft was created. The document was sent to the other members of the research team for comments as well to a small number of panel members who expressed a particular interest in reviewing the draft guidelines. As a result of this feedback, some minor changes were made.

### Ethical approval

The study received ethical approval from the University of Melbourne (in Australia), the University of Palermo (Argentina) and the University of Chile (Chile).

## Results

### Round 1

A total of 54 participants completed the Round 1 questionnaire. The professionals (*N* = 31) were equally distributed from Chile (*N* = 15) and Argentina (*N* = 16), and comprised 14 psychologists, 10 psychiatrists, two occupational therapists, two rehabilitation technicians, one nurse, one general practitioner and one unspecified health professional. The average years of experience as health professional was 21.2 years, with 68% males (*N* = 21) and 32% females (*N* = 10).

The lived experience panel (*N* = 23) were all Argentinian and were mostly from Buenos Aires city (*N* = 15). Eight other participants belonged to four other provinces (Santa Cruz, Rio Negro, Mendoza and San Luis). Twelve experts were consumers and 11 were carers and/or relatives from other consumers. A total of 65% were males (*N* = 15) and 35% were females (*N* = 8). See Table 1. Two experts in Argentina who had been invited for the lived experience panel self-identified as health professionals, volunteered for the other experts’ panel and were consequently moved to the latter panel.

**Table 1:** Sample characteristics

	First round n (%) (*n* = 54)	Second round n (%) (*n* = 45)
Sex		
Female	18 (33.3%)	16 (42.2%)
Male	36 (66.7%)	29 (57.8%)
Profession (professional panel)	31	25
Psychologists	14 (45.1%)	10 (40.0%)
Psychiatrists	10 (32.2%)	9 (36.0%)
Occupational therapists	2 (6.4%)	2 (8.0%)
Rehabilitation technicians	2 (6.4%)	1 (4.0%)
Nurses	1 (3.2%)	1 (4.0%)
General Practitioner	1 (3.2%)	1 (4.0%)
Unspecified health practitioner	1 (3.2%)	1 (4.0%)
Source of experience (lay panel)	23	20
Familial experience or peer support experience	11(47.8%)	9 (45.0%)
Own experience	12 (52.2%)	11 (55.0%)

Out of the 182 statements initially rated by the two panels of experts, 106 items (58.2%) were endorsed as *essential* or *important* by ≥ 80% of the panel members in each of the two groups. Another 33 items required re-rating in Round 2, and 43 items were rejected (Fig. 1). The endorsement rates from the two panels were 70% and 66% for the lived experience and the professional’s panel respectively. A total of 4.4% of items (*N* = 8) were endorsed by one panel and rejected by the other panel, implying a high concordance between panels. See supplementary files 1 and 2 for the endorsement of the Spanish statements divided by panels.

### Round 2

The Round 2 questionnaire included 61 new items suggested by the experts in the Round 1 in addition to the 33 items to be re-rated (Fig. 1). A total of 45 participants completed the Round 2. The professionals’ panel included 25 experts and the lived experience panel included 20 experts, with response rates of 80.6% and 86.9% of the Round 1 participants respectively. No new participants were added in Round 2. In Round 2, items were included when endorsed by one panel with an acceptance rate of 80% or more, and 75% or more by the other panel. Out of the 94 statements rated in the Round 2, 59 items (62.8%) were endorsed by both panels and 35 were rejected. New items received a lower endorsement (54.1%) compared to re-rated items (78.8%).

### Differences between the Spanish-language guidelines for Chile and Argentina and the English-language guidelines

In total, across the two rounds, 165 items were endorsed, and 78 items were rejected. Compared to the English-language guidelines, it is worth noting that 50 statements (27.5%) included in the English guidelines were not accepted by the Argentinian and Chilean experts. This includes: (a) almost every item with regards to understanding low-risk drinking (except for “the first aider should tell the person that changing drinking patterns is difficult, but they should not give up trying”), (b) all practical tips for low-risk drinking, and (c) other salient items from different sections of the guidelines:

Alcohol use problems:

- The first aider should be aware that it is possible for the person to change their drinking habits (on their own).
- The first aider should be aware that problem drinking may be related to an untreated mental illness.
- The first aider should have general knowledge of some of the reasons why people drink alcohol to excess.

Approaching someone about their drinking:

- The first aider should use ‘I’ statements, for example, "I am concerned about how much you’ve been drinking lately".
- The first aider should ask the person about their drinking behaviour, e.g. about how much alcohol the person tends to drink.
- The first aider should discuss with the person the link between their drinking behaviour and the negative consequences.
- The first aider should encourage the person to find some information on how to reduce the harms associated with their problem drinking.
- The first aider should talk to the person when both are in a calm frame of mind.

Professional help:

- If the person is unwilling to get professional help, because they don't want to stop drinking completely, the first aider should explain that the treatment goal may be to reduce alcohol consumption rather than to quit altogether.
- The first aider should explain to the person that there are several approaches available for treating drinking problems.

First aid for alcohol intoxication:

- The first aider should be aware that the body only metabolises approximately one standard drink of alcohol an hour.
- The first aider should be aware that only time will reverse the effects of intoxication.
- If it is unsafe to prevent the person from driving, the first aider should call the police.

First aid for alcohol withdrawal:

- The first aider should seek medical help if the person has been drinking heavily for long periods and decides to stop suddenly.

### Differences between the lived experience and health professional panels

The lived experience panel and the health professionals’ panel were mostly in agreement (r = 0.71), with 65% of items having less than a 10% difference in the percentage of members of the panels endorsing those items, including 7% of items with an absolute agreement on both panels (i.e., 100% of members of both panels endorsing the item). However, there were 13.2% of items where the disagreement between panels was 20% or higher, showing significant differences between the two panels. Notably, almost 5% of the items (*N* = 12) had rating differences of more than 30%. In addition, ratings of consumers and carers were strongly correlated (r = 0.75) in Round 1, supporting the decision to combine them into a single panel of experts with lived experience.

The greatest differences pertained to items suggested by members of the panels in Round 1 and tested in the Round 2. These items were endorsed by 100% of the lived experience panel but were largely not endorsed by the health professionals. These were: “The first aider should know that the treatment an alcoholic person involves completely stopping drinking since their drinking causes damages at a personal, familial and social level”, and “The first aider should know that the alcoholic person needs to be convinced of starting a new way of life to be able to change their drinking behaviour”. These statements were respectively endorsed by 40% and 44% of the members of the health professionals’ panel. The third statement with a large rating difference was that “The first aider should know that the ambivalence towards professional help stems from the person’s fear to change,” with 48% of professionals and 90% of consumers endorsing this item.

With regards to statements included in the Round 1, the most significant difference in rating was in the item: “The first aider should be aware that the person is the only one who can make the decision to change their drinking behaviour,” which was endorsed by 64% of the members of the health professionals’ panel and by 100% of the lived experience panel. Another statement with significant differences between the two panels was that “The first aider should speak with a gentle, caring tone of voice,” which was endorsed by 74% of consumers and barely 41% of health professionals.

## Discussion

This study aimed to develop guidelines for members of the public providing mental health first aid to people with problem drinking in Chile and Argentina. These guidelines comprise 165 statements that were endorsed by both professional and lived experience panels. While 72% of the items included in the English-language guidelines were endorsed, there were some notable differences, particularly those related to abstinence. These differences were greater than the guidelines for problem drinking culturally adapted for China [42] and Brazil [43], where 86% and 84% of English-language items were endorsed, respectively. While there was a relatively high level of agreement between health professionals and people with lived experience, some divergence of opinion was seen, particularly in commitment to recovery as a condition for help.

### No practical tips for low-risk drinking

Unlike previous versions of these guidelines, developed with English speaking international experts as participants, Chilean and Argentinian experts rejected the idea that the first aider should provide information on low-risk drinking to individuals experiencing drinking problems. Notably, less than 20% of experts in both panels endorsed the idea that “If the person wants some advice on low-risk drinking, the first aider inform the person that the number of standard drinks is often listed on the beverage's packaging.” This may be explained by two possibilities, that local experts gave their ratings while considering a person experiencing an alcohol dependency condition rather than milder drinking problems, and that local experts are impacted by the widespread problem of alcohol consumption in the general population in Chile and Argentina and consider that abstinence is the best way to tackle alcohol drinking problems. Moreover, in Chile and Argentina, the health promotion approach to alcohol tends to focus on abstinence rather than harm reduction. The health professional panel members were also unlikely to endorse items related to low-risk drinking. Moreover, many of the lived experience participants, who were influenced by AA tenets, endorsed these items on average 10% less often than professionals, possibly due to the belief that suggesting low-risk drinking may make the person believe that they are authorized to drink [42, 46].

### Approaching to someone with drinking problem in Chile and Argentina

While most of the suggestions accepted in Australia on how to approach someone with drinking problems were accepted by the local experts, a few rejected items stand out as specific to Chile and Argentina. A direct approach to the person, as exemplified by items relating to asking the person about their drinking and discussing negative consequences, was rejected, as were the items about how to engage with the person (e.g., using ‘I statements’ and waiting for the person to be calm). This may be explained by the overarching interpersonal cultural orientation common in Latin American society, in which being open, warm and attuned to the wishes and feelings of others is highly valued [47]. Thus, a direct or confrontational approach may be considered inappropriate.

### The importance of committing to recovery

The experts did not agree on the importance of the person with drinking problems needing to be convinced of their desire for a different life without alcohol as crucial for seeking for help and making a change in their drinking patterns. While this was unanimously accepted by experts with lived experience, professionals cast significant doubts on its importance. Experts from the professional panel are likely to believe that they can help someone with drinking problems to commit to recovery even if they are in a different mindset when they first seek help. On the other hand, lived experience experts may have drawn from their own experience of crises (i.e., “bottoming out”) as well as from their experience in AA. The reluctance of local health professionals to accept that individuals with mental health problems may have a role in self-directing their recovery process [48, 49] may explain why the experts from the professional panel could have overestimated their capacity to help someone who is unwilling to seek help for their drinking problems. In the same vein, the health professional experts were less likely than experts with lived experience to agree on the importance of the first aider suggesting that the person avoids places where alcohol will be available, and that the person only attends such places after a time of sobriety or accompanied by someone who is aware of their drinking problems.

### Lived experience experts are rarely accorded equal status with health professionals

In this study, lived experience experts and health professionals had the same status and their opinions were equally considered. It is uncommon in Argentina and Chile to seek the opinions of people with lived experience (either their own or as support people) [50, 51]. Several participants from this panel expressed gratitude for being asked about their opinions and showed clear appreciation for the MHFA project. However, it is also important to mention that some participants invited because of their history as consumers or caregivers agreed because of their lived experience but answered the questionnaires by signing as health professionals. Such affiliation change may indicate discomfort in the role of a user or a caregiver, possibly due to stigma leading to a preference to identify as a health professional.

### Strengths and limitations

Strengths of the study include the involvement of people with lived experience of problem drinking, the high retention of participants from both panels across survey rounds and the evaluation of many new items suggested by the local panels. This has allowed us to develop culturally sensitive guidelines to help individuals with problem drinking in Chile and Argentina. However, a few limitations need to be highlighted. The lived experience panel had no representatives from Chile; however, cultural similarities prevail (e.g., Catholicism, Hispanic roots, alcohol as a significant domestic problem). Moreover, there was a high degree of agreement between health professionals from Argentina and Chile, which suggests that responses from individuals with lived experience in Chile would likely be similar to those in Argentina. Experts may have given their ratings while considering a person experiencing an alcohol dependency condition rather than milder drinking problems and experts with an AA background may have influenced the rejection of most low-risk drinking statements.

## Conclusion

Through this Delphi expert consensus study, we created mental health first aid guidelines for problem drinking applicable to the Chilean and the Argentinian populations. These guidelines provide a range of mental health first aid strategies, such as how to communicate with a person with problem drinking, what to do if the person is intoxicated, and how to deal with emergencies related to alcohol intoxication and alcohol withdrawal. The widespread rejection of understanding and offering advice on low-risk drinking stands out as a significant difference to the English-language guidelines. The ratings of health professionals and individuals with lived experience were mostly highly correlated. However, there were considerable differences in the commitment to recovery as a condition for help. The guidelines may be used as a standalone product and may also be used to inform MHFA training for Argentina and Chile. Future research should assess the effectiveness of the first aid strategies endorsed within these guidelines to ensure that mental health literacy and access to care are improved and that stigma is reduced.

## Supplementary Information

**Additional file 1.** Guidelines for Chile and Argentina.

**Additional file 2. **Statements that were presented to the panels and their ratings across 2 rounds of the survey.

## Data Availability

The data supporting our findings is attached as the Additional file, which contains all the statements that were presented to the panels and their endorsement rates.

## Abbreviations

MHFA: Mental Health First Aid
AA: Alcoholics Anonymous
GIA: Alcoholism Institutional Groups [Grupos Institucionales de Alcoholismo]
