# Supplementary material for: Development of mental health first aid guidelines for problem drinking: a Delphi expert consensus study in Argentina and Chile
Source: BMC Psychiatry. 2022 Feb 12;22:113. doi: 10.1186/s12888-022-03749-x (PMC8841054; doi:10.1186/s12888-022-03749-x)
Supplement: Supplementary file 2 — Additional file 2. Statements that were presented to the panels and their ratings across 2 rounds of the survey. [file 12888_2022_3749_MOESM2_ESM.pdf]

MENTAL HEALTH FIRST AID
Chile - Argentina

CÓMO AYUDAR A UNA PERSONA QUE EXPERIMENTA PROBLEMAS EN EL CONSUMO DE ALCOHOL

GUIA DE PRIMEROS AUXILIOS EN SALUD MENTAL

¿Cuáles son los problemas en el uso del alcohol?

El uso problemático del alcohol se refiere a consumir alcohol al punto en que se le asocian daños de corto y/o de largo plazo. Dichos daños incluyen:

• Problemas familiares o problemas sociales (por ejemplo, problemas familiares o divorcios, pérdida de trabajos, problemas económicos, etc.)

• Accidentes no fatales o, directamente, fatales (por ejemplo, accidentes de auto, caídas, episodios de violencia, etc.)

• Problemas de salud mental (por ejemplo, depresión, ansiedad, problemas para dormir, etc.)

• Problemas de salud general (por ejemplo, problemas en el hígado, cardíacos, úlceras estomacales, etc.)

Por lo general se acepta que las personas recurren al consumo de alcohol por una variedad de causas y, posiblemente, en conexión con diferentes tipos de trastornos mentales. Dichos trastornos (por ejemplo, la depresión) pueden requerir un abordaje adicional y se sugiere consultar también esas guías específicas de primeros auxilios.

Tipos de problemas en el uso del alcohol

De acuerdo a consideraciones y clasificaciones de la Organización Mundial de Salud, una persona se considera que tiene un problema en el uso del alcohol si se encuentra dentro de las siguientes categorías:

1. Consumo de riesgo: se refiere al consumo de alcohol que aumenta el riesgo de las consecuencias adversas. La Organización Mundial de la Salud (OMS) lo describe como el consumo diario de alcohol de 20 a 40 grs para las mujeres, y de 40 a 60 gramos para los hombres. Los gramos de alcohol en una bebida alcohólica varían por país. Por ejemplo, en Chile, se ha estimado que en promedio una bebida tiene alrededor de 15 grs.

2. Consumo perjudicial: se refiere al consumo regular de alcohol que se asocia a consecuencias negativas para la salud de las personas. La organización mundial de salud establece el consumo perjudicial de 40 grs al día para las mujeres y 60 grs al día para los hombres.

3. Dependencia al alcohol: conjunto de fenómenos del comportamiento, cognitivos y fisiológicos que pueden desarrollarse tras el consumo repetido de una sustancia (por ejemplo, reacción de abstinencia física en ausencia de la sustancia). La dependencia al alcohol está tipificada en el manual de Clasificación Internacional de Enfermedades de la OMS (CIE) actualmente en su versión 11. El diagnóstico de dependencia a una sustancia (puede ser alcohol u otra sustancia) requiere de tres o más de los criterios especificados en el plazo de un año.

CÓMO AYUDAR A UNA PERSONA QUE EXPERIMENTA PROBLEMAS EN EL CONSUMO DE ALCOHOL

4. Trastorno en el uso del alcohol: involucra dos o más de los siguientes puntos en alguno de los últimos 12 meses:

- Tomar grandes cantidades o en períodos más prolongados de lo que se intentaba
- Problemas para reducir o controlar el consumo de alcohol
- Pasar mucho tiempo buscando, consumiendo o recuperándose del consumo de alcohol
- Sintiendo un deseo intenso de beber
- Pérdida de actividades importantes de la esfera social, laboral o recreativa debido a problemas con el consumo de alcohol
- Consumo repetido del alcohol aun cuando eso le causa problemas (a sí mismo y/o en su relación con otros)
- Consumo repetido incluso en situaciones que pueden acarrear un peligro
- Síntomas de abstinencia cuando intenta parar o disminuir el consumo de alcohol
- Aumento de la tolerancia al consumo del alcohol (por ejemplo, para sentir los mismos efectos la persona debe consumir más alcohol)
- Sigue tomando a pesar de ya conocer o haber experimentado los efectos perjudiciales de tomar

Referencias:

Anderson, P., Gual, A., & Colon, J. (2008). Alcohol y atención primaria de la salud: informaciones clínicas básicas para la identificación y el manejo de riesgos y problemas. Organización Panamericana de la Salud.

Leyton, F. F., & Arancibia, P. (2016). El consumo de alcohol en Chile: situación epidemiológica. Santiago de Chile, Ministerio de Salud, SENDA Ministerio del Interior y Seguridad Pública, Gobierno de Chile.

Organización Mundial de la Salud (2019). Clasificación Internacional de Enfermedades. OMS. Disponible en https://icd.who.int/es

CÓMO AYUDAR A UNA PERSONA QUE EXPERIMENTA PROBLEMAS EN EL CONSUMO DE ALCOHOL

GUIA DE PRIMEROS AUXILIOS EN SALUD MENTAL

MENTAL HEALTH FIRST AID Chile - Argentina

El problema con la bebida

Es importante que tengas en cuenta que existen diferentes formas de consumo de alcohol. Por lo tanto, debes ser capaz de reconocer el consumo problemático del alcohol de aquel consumo que no lo es. Dado que la persistencia del hábito del consumo de alcohol aumenta los riesgos de consecuencias adversas para la salud, es importante que puedas ser capaz de reconocer los signos de consumo de riesgo. Asimismo, debes poder reconocer el consumo perjudicial y los síntomas de la dependencia del alcohol. Las definiciones de la OMS y las guías locales pueden ayudarte a conocer las diferencias. Es importante poder identificar cuándo la persona requiere una atención especializada para sus problemas con la bebida. Sin embargo, es bueno que tengas presente que los expertos con experiencia vivida en el uso de alcohol han advertido que no sería importante la indagación por las razones por las cuales las personas toman alcohol en exceso.

La persona que tiene un problema en el consumo de alcohol puede no reconocerlo, puede incluso negarlo cuando es confrontado y puede generar una situación de conflicto al intentar que tome conciencia del problema que tiene. Además, es probable que la persona con problemas de consumo de alcohol no desee hablar del problema al realizar una consulta profesional.

La fuerza de voluntad de la persona no suele ser suficiente para ayudarla a detener el consumo problemático del alcohol en tanto no es fácil cambiar los hábitos de consumo y tampoco es esperable que la persona modifique sus hábitos de consumo por sí misma. La persona puede recaer una o varias veces antes de cambiar sus patrones de consumo. Los consejos no suelen ser muy útiles para lograr que una persona modifique su consumo de alcohol. La desintoxicación es tan solo una parte del proceso de recuperación y se suelen requerir muchos cambios en el estilo de vida para cambiar los comportamientos de consumo de alcohol. Enfermarse por el consumo de alcohol lleva tiempo y suele llevar tiempo desandar ese proceso. Sin embargo, es posible ayudar a la persona a cambiar su problema con la bebida ya que el consumo perjudicial y la dependencia al alcohol son un problema que tiene tratamiento adecuado. Aun así, las personas pueden no estar informadas al respecto.

La persona puede estar bebiendo como un medio para hacer frente a los problemas en su vida (por ejemplo, angustia subyacente u otro tipo de trastorno mental), es mejor que no indagues sobre eso para evitar que la persona encuentre razones para seguir bebiendo.

Además, los problemas de salud mental pueden ser causados o exacerbados por el consumo de alcohol. En tanto la dependencia al alcohol es una enfermedad en sí misma, hay que ser muy cauto a la hora de suponer que el problema con el alcohol puede estar relacionado con una enfermedad mental no tratada o que primero haya que abordar la angustia emocional subyacente. La dependencia al alcohol requiere ser abordado inmediatamente y sin dilación.

Es importante aconsejar a la persona para que identifique las situaciones en las que es más probable que beba y evitarlas de ser posible. La red social donde se encuentra inserta la persona puede ser clave para comprender su consumo de alcohol. Igualmente, es importante detectar eventuales apoyos en la familia o grupos de pertenencia, e incluirlos en el abordaje. Hay que alentar a la persona a comunicarse con amigos y familiares que apoyen sus esfuerzos para cambiar sus conductas con la bebida. Asimismo, hay que advertir a la persona que no todos los familiares y amigos apoyarán sus esfuerzos para cambiar su comportamiento con respecto al consumo de alcohol.

A menudo hay presión social para emborracharse cuando se bebe. Es importante decirle a la persona que tiene derecho a rechazar el alcohol cuando se sienta presionada para beber, y que puede decir “no, gracias”, sin explicación, cuando se sienta esta presión. Llegado el caso, hay que alentar a la persona a practicar diferentes formas de decir “no” a la presión social para beber. Hay que alentar a la persona a pasar tiempo con familiares y amigos que no beben, que puedan apoyar el esfuerzo de la persona para cambiar su comportamiento de consumo de alcohol.

No hay acuerdo entre los expertos respecto de si es necesario que la persona con problemas de consumo de alcohol esté convencida de desear comenzar un cambio en su forma de vida para poder cambiar. Esto es fundamental de acuerdo a las personas con experiencia vivida, aunque no para los profesionales de la salud.

CÓMO AYUDAR A UNA PERSONA QUE EXPERIMENTA PROBLEMAS EN EL CONSUMO DE ALCOHOL

GUIA DE PRIMEROS AUXILIOS EN SALUD MENTAL

Un primer paso para ayudar a una persona con problemas con el consumo de alcohol puede ser lograr que consulte con un médico para evaluar su estado clínico y contar con un análisis de laboratorio para conocer el compromiso orgánico. Es importante poder tener acceso a un especialista con quien resolver dudas acerca de cómo ayudar a la persona. La ingesta excesiva de alcohol suele estar acompañada de otras dependencias (tabaco, psicofármacos, otro tipo de sustancias, juego).

Es importante que conozcas los aspectos vinculados a los derechos de niños/as y adolescentes en caso de que la persona fuera menor de edad.

Cómo hablar con una persona sobre su consumo de alcohol

Cuando hables con una persona que tiene problemas con el consumo de alcohol, se debe dejar en claro qué es lo que como asistente estás dispuesto a hacer o qué cosas son posibles de hacer para ayudarla. Dentro de las actividades que un asistente puede realizar se encuentran acciones que van desde escuchar hasta organizar la obtención de ayuda profesional, pero no es el asistente quien podrá brindar el tratamiento a la persona.

La persona debe ser consciente de que no se le obligará a obtener ayuda profesional, excepto en ciertas circunstancias, por ejemplo, si un incidente violento hace que se llame a la policía o si la persona se encuentra en una emergencia médica.

Es importante hablar con la persona en un ambiente tranquilo y privado; interactuar con la persona de una manera contenedora, sin hacer uso de una modalidad confrontativa en la medida de lo posible, y tratar de escuchar a la persona sin juzgarla. Se recomienda a su vez, evitar juicios morales sobre el consumo de alcohol de la persona o “acusar” a la persona de “ser alcohólica”. En ningún caso hay que usar un enfoque amenazador. Tampoco se debe adoptar una actitud como si se le fuese a dar una clase a la persona. Al momento de hablar con una persona sobre su consumo, puede ser de ayuda identificar y discutir el comportamiento de la persona en lugar de criticarla (por ejemplo, se le puede decir: “Parece que tu forma de beber te está trayendo problemas con tus amistades”, en lugar de decirle “Lo que haces esta mal”). Además, es importante que tengas una actitud empática y colaborativa, de escucha activa; es decir, centrar la atención completamente en el otro para identificar lo que la persona quiere decir y lo que le está ocurriendo (por ejemplo, se puede adaptar qué decir y cómo hablar en función del estadío de motivación para el cambio de la persona, o resumir lo que se entendió de lo que la persona dijo para corroborar si es correcto).

Para hablar con la persona acerca de su consumo de alcohol se recomienda hacerlo de manera abierta y honesta, tratando de entender la percepción que la persona tiene de su consumo de alcohol. Es importante preguntar a la persona si cree que su consumo de alcohol es un problema.

Cuando se está en interacción con una persona que tiene un problema asociado al consumo de alcohol es esperable que ella no muestre un cambio en el pensamiento o el comportamiento de la persona de inmediato, o que no quiera cambiar su comportamiento en absoluto; esta conversación puede ser la primera vez que la persona piensa (o se da cuenta) que su consumo de alcohol es un problema.

Es importante tener presente que la persona con la que converses es un ser humano con historia, identidad, ideas, sentimientos, y temores, y nunca es solamente un “alcohólico”. Se debe evitar etiquetar a la persona (por ejemplo, un “alcohólico” o “adicto”) ni asociarla a características negativas (como “violento”, “delincuente”, u otras) que promuevan el estigma.

Debes considerar la disposición de la persona para hablar sobre las áreas de su vida que podrían estar siendo afectadas por el consumo de alcohol (por ejemplo, su estado de ánimo, el desempeño laboral y las relaciones con otros).

Es importante que tengas presente que la persona puede no recordar los eventos que ocurrieron mientras estaba intoxicada (es decir, que podría haberse desmayado).

Es importante que informes a la persona que el alcohol puede interactuar con otras drogas (ilícitas o recetadas) de una manera impredecible y que puede llevarla a una emergencia médica.

Es importante tener presente que se puede recurrir a un profesional de la salud para determinar la mejor manera de acercarse a la persona para encarar aquello que resulte preocupante de la situación de la persona.

CÓMO AYUDAR A UNA PERSONA QUE EXPERIMENTA PROBLEMAS EN EL CONSUMO DE ALCOHOL

GUIA DE PRIMEROS AUXILIOS EN SALUD MENTAL

Si bien algunas guías incluyen la información sobre consumo saludable de alcohol que podría ofrecer el asistente de primeros auxilios a las personas, los expertos de Argentina y de Chile opinaron que esta información no es recomendable que le sea provista a la persona. Sin embargo, los tipos de problema en el consumo de alcohol tal como los ha definido la OMS pueden ser útiles sin que ello implique que quienes tienen problemas más severos con el alcohol puedan beneficiarse por el hecho de simplemente conocer estas categorías.

La ayuda profesional

Las siguientes señales de advertencia indican que la persona necesita ayuda profesional:

• La persona reconoce que piensa mucho sobre el alcohol y cuándo tendrá la oportunidad de beber.

• La persona tiene deudas debido a la cantidad de dinero que gasta en alcohol.

• La persona se vuelve ansiosa cuando no puede acceder al alcohol.

• La persona necesita alcohol para lidiar con ciertas situaciones.

• La persona se mete en discusiones o tiene accidentes debido al alcohol.

• La capacidad de la persona para realizar tareas cotidianas se ve gravemente afectada.

• La persona ha faltado a sus actividades o compromisos por haber bebido alcohol en exceso (por ejemplo, dejar de ir al trabajo).

• La persona ha intentado disminuir o dejar de consumir, pero no lo ha logrado.

• La persona no puede detener su consumo de alcohol una vez que comienza a beber.

• La persona experimenta diversos síntomas adversos físicos cuando no ha bebido.

• La persona que toma ejerce violencia verbal o física contra sus convivientes.

• El alcohol le trajo problemas legales.

• Toma ni bien se levanta de dormir.

Es importante que informes a la persona sobre la gama de apoyos no profesionales disponibles para el consumo de alcohol, por ejemplo, grupos de ayuda mutua.

Es posible que la persona no desee ayuda profesional cuando se le sugiere por primera vez, y puede ser que le resulte difícil aceptar ayuda profesional.

Hay que asegurarle a la persona que la ayuda profesional es confidencial y decirle a la persona que se la apoyará para obtener ayuda profesional. Si la persona está dispuesta a buscar ayuda profesional, hay que darle información sobre las opciones disponibles y alentar a la persona a hacer una cita.

Si la persona NO está dispuesta a obtener ayuda profesional, es fundamental que:

• Seas compasivo y paciente mientras se espera que la persona acepte que la necesita.

• Estés preparado para hablar con ella nuevamente en el futuro.

Es importante que continues sugiriendo la ayuda profesional a la persona si ésta se pone a sí misma o a otros en riesgo.

Cambiar los hábitos de bebida/dejar de beber es difícil, pero es importante transmitir a la persona que no debe dejar de intentarlo.

Si la persona se ha comprometido a realizar una consulta profesional verifica que la misma haya tenido lugar y muestra interés por lo ocurrido. Si la persona inició un tratamiento, en la medida de las posibilidades, refuerza la idea de la continuidad de las consultas y el cumplimiento de los tratamientos indicados.

Es habitual la existencia de ambivalencia hacia la ayuda profesional tanto en la persona con problemas de consumo de alcohol, como en sus grupos de pertenencia.

Si una persona no quiere cambiar su comportamiento, y eres parte de su círculo cercano, NO deberías:

• Tomar junto con ella.

CÓMO AYUDAR A UNA PERSONA QUE EXPERIMENTA PROBLEMAS EN EL CONSUMO DE ALCOHOL

GUIA DE PRIMEROS AUXILIOS EN SALUD MENTAL

• Tratar de controlarla mediante el soborno, regañando, amenazando o llorando con ella.

• Sentirte culpable o responsable.
• Encubrir o dar excusas.

• Asumir las responsabilidades de la persona, excepto si no hacerlo podría causar daño (por ejemplo, a su propia vida o la de otra persona).

• Limitar la ayuda a la que le pueda dar sino más bien tratar de buscar otro asistente que pueda ayudar a su familiar o allegado.

Los cambios pueden ser transitorios y, cuando se vuelve a los hábitos anteriores, esto puede ser fuente de frustración tanto para la persona como para su grupo de pertenencia.

Existen barreras en el acceso a servicios de salud para personas con alcoholismo y la persona puede enfrentarse a situaciones de rechazo o de desconocimiento acerca de cómo atenderlas.

Los primeros auxilios para una persona intoxicada

Es importante que puedas reconocer los síntomas y señales de que una persona está intoxicada y requiere ayuda médica pronta o si es una urgencia. No tengas miedo de buscar ayuda médica para la persona intoxicada, si es necesario, incluso si puede haber implicaciones legales para la persona intoxicada.

Algunos signos de intoxicación por alcohol son:

• Confusión o estupor (estar conciente pero sin responder)

• Dificultades para hablar

• Pérdida de coordinación de movimientos

• Vómitos

• Convulsiones

• Respiración lenta o irregular

• Respiración irregular (intervalos de más de 10 segundos entre respiraciones)

• Piel azulada o pálida

• Hipotermia (temperatura corporal entre 33 y 35 grados celsius)

• Pérdida del conocimiento

Referencias:

https://www.mayoclinic.org/es-es/diseases-conditions/alcohol-poisoning/symptoms-causes/syc-20354386

https://www.nhs.uk/conditions/alcohol-poisoning/

Es fundamental mantener la calma. A pesar de que la persona puede no lograr comprender todo lo que se le dice y expresarse con dificultad, hay que hablar con la persona intoxicada de manera respetuosa. Nunca reírse, burlarse ni provocar a la persona intoxicada; usar un lenguaje simple y claro. La persona intoxicada puede reaccionar de forma exagerada a las palabras negativas; por lo tanto, hay que usar palabras positivas (como “trata de mantener la calma”) en lugar de palabras negativas (como “no debes pelear”). En ese momento no tiene sentido intentar involucrar a la persona en una conversación profunda sobre su comportamiento de bebida si está intoxicada.

Es fundamental conocer en qué situaciones de intoxicación de una persona no deberías actuar solo. Hay que conocer los teléfonos o forma de acceder a la red de urgencia local para la gestión o derivación de una persona intoxicada.

Se debe permanecer con la persona intoxicada o hay que asegurarse de que no se quede sola. Es importante que sepas que beber café negro, dormir, caminar y duchas frías no revertirán los efectos de la intoxicación.

Se deben hacer arreglos para que la persona intoxicada vaya a un hospital si la persona se encuentra en riesgo vital. Hay que recordar que la intoxicación puede llevar a una emergencia médica, y por tanto, debes preguntar a la persona intoxicada si ha tomado algún medicamento u otra droga, en caso de que el estado de la persona empeore y derive en una emergencia méd-

ica. La persona intoxicada por su mismo estado puede no estar en condiciones de brindar información importante por lo que es conveniente que un amigo o miembro de la familia acompañe a la persona intoxicada al hospital, ya que puede proporcionar dicha información relevante.

La persona puede estar más intoxicada de lo que aparenta, y hay que recordar que el consumo de alcohol puede enmascarar el dolor de lesiones que pudiera haber tenido en el contexto de la intoxicación.

La intoxicación por alcohol es un proceso muy dinámico y cambiante que puede llevar a que el cuadro empeore rápidamente.

Debes evaluar la situación para detectar posibles peligros y asegurarse que la persona intoxicada y otros estén a salvo. Hay que asegurarse de que la persona intoxicada esté lejos de máquinas y objetos peligrosos. También, tener en cuenta que cuando está intoxicada, la persona puede participar en una amplia gama de actividades riesgosas, como tener relaciones sexuales sin protección, destrozar bienes o conducir un automóvil. Se debe desalentar a la persona intoxicada de conducir un vehículo o andar en bicicleta, por ejemplo, informando sobre los riesgos tanto para ella como para otros. Hay que hacer todo lo posible por evitar que la persona intoxicada conduzca un vehículo o monte en bicicleta y organizar un modo seguro de transporte para que la persona intoxicada llegue a casa.

Si la persona intoxicada expresa pensamientos suicidas (por ejemplo, declara que desea suicidarse) o demuestra un comportamiento suicida (por ejemplo, trata de caminar delante de los autos), llama a los servicios de emergencia para obtener ayuda.

Si la persona intoxicada no tiene pulso, hay que tener presente que se necesita realizar una resucitación cardiopulmonar (RCP). Hay que mantener abrigada a la persona intoxicada para prevenir la hipotermia (teniendo en cuenta que, aunque la persona puede sentir calor, la temperatura de su cuerpo puede estar disminuyendo). Si la persona ha vomitado, se deben despejar las vías respiratorias de la persona si es necesario.

Si la persona está vomitando y consciente, se debe mantener a la persona sentada o ponerla en posición de recuperación. Si la persona está vomitando e inconsciente, se debe colocarla en la posición de recuperación y permanecer con ella hasta que llegue la ayuda médica. Se debe tener siempre presente que si la persona se queda acostada sobre su espalda, podría ahogarse con su vómito o su lengua podría bloquear sus vías respiratorias.

Si la persona intoxicada es difícil de despertar, hay que ponerla en posición de recuperación. Si la persona intoxicada muestra signos de una posible lesión en la cabeza (por ejemplo, está vomitando y hablando incoherentemente), hay que buscar ayuda médica inmediata.

La intoxicación aguda por alcohol, es un nivel de intoxicación que puede llevar a la muerte. La cantidad de alcohol que causa una intoxicación aguda por alcohol es diferente para cada persona. Se debe llevar a la persona intoxicada al departamento de emergencias de un hospital si existe la preocupación de que su nivel de intoxicación sea peligroso.

Se debe llamar a una ambulancia en cualquiera de los siguientes casos:

- la persona vomita continuamente
- no puede ser despertada
- la respiración es irregular, superficial o lenta
- la frecuencia del pulso es irregular, débil o lenta
- la piel de la persona intoxicada es fría, húmeda, pálida o azulada
- la persona cae inconsciente.

No hay que esperar a que todos los síntomas de la intoxicación aguda por alcohol estén presentes para obtener ayuda médica. Si se sospecha de intoxicación aguda por alcohol, hay que asegurarse de que la persona intoxicada no se quede sola, llamar a una ambulancia de inmediato, y no darle comida (ya que pueden ahogarse con ella si no está completamente consciente).

Es imprescindible que garantices tu propia seguridad en todo momento. En tal sentido, se debe evitar la escalada de la confrontación lo más posible o discutir con la persona intoxicada. Hay que permanecer lo más tranquilo posible, hablar despacio y con confianza, evitando manifestarse de una manera hostil o amenazante.

CÓMO AYUDAR A UNA PERSONA QUE EXPERIMENTA PROBLEMAS EN EL CONSUMO DE ALCOHOL

GUIA DE PRIMEROS AUXILIOS EN SALUD MENTAL

Es importante considerar hacer pausas en la conversación para permitir que la persona intoxicada tenga la oportunidad de calmarse y hay que prestar atención a signos de agresión creciente de parte de la persona intoxicada. Si ha habido violencia, se debe buscar la asistencia de emergencia apropiada.

La posición de recuperación

Una persona inconsciente necesita atención médica inmediata y que sus vías de respiración se conserven despejadas. Si la persona permanece acostada boca arriba puede atorarse con su propio vómito o su lengua podría impedir el ingreso de aire. También es conveniente verificar que no hubiera vidrios rotos o algún otro tipo de objeto punzante con el que la persona podría lastimarse al ser puesta en posición de recuperación.

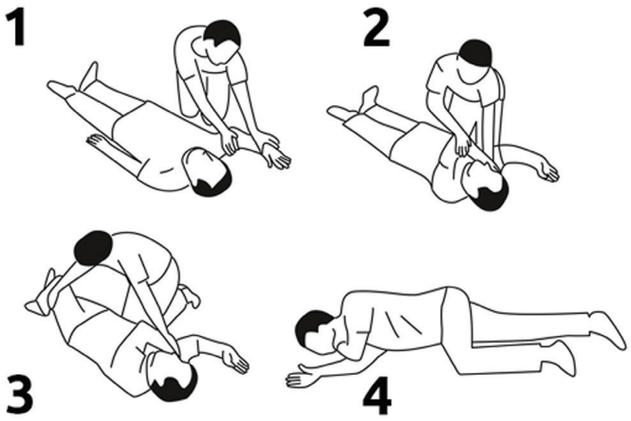

La persona en abstinencia de alcohol

Es importante que sepas qué es la abstinencia de alcohol y que seas capaz de reconocer los síntomas de la abstinencia de alcohol.

Se refiere a los síntomas que puede tener una persona que ha estado consumiendo alcohol en exceso regularmente y que de pronto deja de beber alcohol. Esto ocurre porque físicamente la persona se ha acostumbrado a un nivel base de alcohol, lo que tiene un efecto depresor en el cerebro y otras partes del cuerpo.

Los síntomas típicos incluyen:

- ansiedad
- irritabilidad
- latidos acelerados
- sudor excesivo
- náuseas y vómitos
- insomnio
- hipervigilancia
- pérdida del apetito
- dolor de cabeza

Síntomas que indican gravedad son:

- fiebre
- disminución del ritmo respiratorio
- alteraciones del ritmo cardíaco
- alucinaciones
- convulsiones
- delirios

Referencia:

Oviedo, Heidi Celina; Arboleda, Patricia Liliana. Fisiopatología y tratamiento del síndrome de abstinencia de alcohol. Universitas Médica [en línea]. 2006, 47(2), 112-120 [fecha de consulta 1 de Junio de 2021]. ISSN: 0041-9095.

Disponible en: *https://www.redalyc.org/articulo.oa?id=231018675002*

Hay que recordar que la abstinencia de alcohol sin medicamentos puede provocar convulsiones. La abstinencia alcohólica con síntomas tipo delirium tremens (delirios) debe ser tratada exclusivamente en un centro asistencial de salud.

MENTAL HEALTH FIRST AID Chile - Argentina

CÓMO AYUDAR A UNA PERSONA QUE EXPERIMENTA PROBLEMAS EN EL CONSUMO DE ALCOHOL

GUIA DE PRIMEROS AUXILIOS EN SALUD MENTAL

Propósito de esta guía

Esta guía de primeros auxilios en salud mental para personas con problemas en el consumo de alcohol está destinada a miembros de la comunidad sin ningún tipo de experiencia o estudio previo referido a la salud / salud mental o el consumo de alcohol. El rol de la persona capacitada por medio de esta guía es poder ofrecer una ayuda inicial a la persona adulta con problemas en el consumo de alcohol hasta cuando ésta reciba la ayuda profesional en el caso de ser necesaria.

¿Cómo se desarrolló esta guía?

El desarrollo de esta guía fue realizado en base a la opinión de expertas y expertos de la Argentina y de Chile, tanto en cuanto a su formación profesional (médicos generalistas, psiquiatras, psicólogos, psicólogos sociales, técnicos de rehabilitación, terapeutas ocupacionales, enfermos, etc.) como personas con experiencia vivida de recuperación de abuso y dependencia de alcohol, o como personas que han provisto apoyo personal a personas expuestas al consumo problemático del alcohol.

Originalmente, el equipo de Argentina y de Chile partió de las guías de primeros auxilios existentes en Australia y realizó la adaptación correspondiente con el asesoramiento de expertos locales.

Los detalles de la metodología empleada pueden consultarse en la publicación Agrest M, Tapia-Muñoz T, Encina E, Wright J, Ardila-Gómez S, Alvarado R, Leiderman E, Reavley N. Development of mental health first aid guidelines for problem drinking: a Delphi expert consensus study in Argentina and Chile. (en proceso de publicación).

¿Cómo usar esta guía?

Esta guía consta de una serie de recomendaciones sobre cómo es posible ayudar a alguien que presenta problemas en el consumo del alcohol. Dado que cada persona es única, es fundamental adaptar a cada una de ellas este conjunto de recomendaciones para poder brindarle la mejor ayuda posible. En este sentido, es posible que algunas personas no puedan ser ayudadas aun siguiendo estas recomendaciones. Debe considerarse muy especialmente que algunas personas presentan junto con el problema en el consumo del alcohol otro tipo de trastornos o enfermedades que pueden requerir abordajes específicos y especializados.

El consumo problemático del alcohol puede suceder en el contexto del uso de otras drogas y/o de otro tipo de problemas de salud mental. A su vez, el consumo muy elevado de alcohol tiene una asociación muy significativa con comportamientos suicidas. Por tal motivo, las guías de primeros auxilios de salud mental correspondientes a Depresión y a Riesgo de Suicidio son muy importantes para consultar por quien se proponga brindar primeros auxilios a alguien que consume alcohol de modo problemático.

Estas guías fueron desarrolladas por la Universidad de Palermo, la Universidad de Chile y la Universidad de Melbourne y están protegidas por el derecho de autor. Sin embargo, se pueden usar libremente si no es con fines de lucro. Se recomienda citar como: Primeros Auxilios en Salud Mental, América Latina. Brindando ayuda a una persona con problemas en el consumo de alcohol: Guía de primeros auxilios en salud mental. Universidad de Palermo, Universidad de Chile y Universidad de Melbourne, 2021.

Si tiene dudas respecto de su utilización puede dirigirse a:

Martín Agrest, magrest66@gmail.com
Thamara Tapia, thammytapia@gmail.com
Nicola Reavley, nreavley@unimelb.edu.au. +61 3 9035 7628

Algunos términos utilizados en esta guía y conceptos asociados

Abuso de alcohol

El abuso de alcohol es un patrón de beber demasiado alcohol con demasiada frecuencia que interfiere con la vida diaria. En ocasiones, el abuso toma la forma de un consumo episódico excesivo o atracón (binge drinking).

El término "abuso" se utiliza a veces con desaprobación para referirse a cualquier tipo de consumo, particularmente, de drogas ilegales, y ha recibido diferentes cuestionamientos para su utilización. Sin embargo, la OMS y el Manual de Diagnósticos y Estadísticas de los Trastornos Mentales (DSM) conservan la denominación.

Alcoholismo

El término fue acuñado originalmente en 1849 por Magnus Huss. Hasta la década de 1940 hacía referencia principalmente a las consecuencias físicas del consumo masivo y prolongado del alcohol. Si bien el término continúa siendo utilizado, la OMS lo dejó de lado en 1980 y pasó a utilizar el de síndrome de "dependencia del alcohol", que es uno de los muchos problemas relacionados con el alcohol. De acuerdo a la Clasificación Internacional de Enfermedades (CIE) en su versión 10, de la OMS, los términos para referirse al consumo problemático del alcohol serían: Intoxicación por alcohol, Dependencia del Alcohol, Abuso de alcohol, Abstinencia de alcohol, así como trastornos relacionados con la intoxicación o la abstinencia (como el delirium o el trastorno psicótico), o derivados del simple consumo de alcohol (como la ansiedad, la depresión o el sueño).

Dependencia del alcohol

La definición técnica del término se refiere al intenso deseo de consumir pese a las consecuencias para el sujeto y a la pérdida de la capacidad de control del uso.

Estigma

Es un fenómeno social que comprende la identificación, separación, pérdida de estatus y discriminación de un grupo de individuos en base a una característica física, psíquica o social. En la identificación, se realiza una distinción de un grupo de individuos en base a características físicas, psicológicas, sociales o culturales (en este caso, "las personas alcohólicas"); en la separación se asocian esas características a creencias negativas (por ej., "las personas alcohólicas son violentas"), y se realiza una distinción entre "ellos" ("los alcohólicos") y "nosotros" ("los no alcohólicos"). Este fenómeno produce una pérdida de estatus o valoración social y se adopta la conducta de discriminación (por ej., "las personas alcohólicas no responden en el trabajo, así que no las contrato", "las personas alcohólicas son violentas, yo no lo soy, no soy amigo de una persona alcohólica").

En conexión con el problema del estigma, en esta guía no usamos el término "persona alcohólica" y destacamos su carácter controversial. Se ha observado, por un lado, que es un término porque ya está socialmente asociado a características negativas y que genera discriminación. Simultáneamente, es un término adoptado por la comunidad de Alcohólicos Anónimos (AA) y con el cual las propias personas con experiencia vivida de esta comunidad eligen nombrarse. En consideración de ambas situaciones, en esta guía hemos decidido utilizar otras formas de denominar a quienes tienen problemas en el uso del alcohol sin dejar de respetar el valor y la importancia que puede tener en la comunidad de AA la adscripción al término "alcohólico".

Intoxicación alcohólica

Estado posterior al consumo excesivo de alcohol, que causa alteraciones en el nivel de conciencia, en lo cognitivo, en la percepción, en el juicio, en la afectividad o en el comportamiento, o en otras funciones y respuestas psicofisiológicas.

Posición de recuperación

Posición recomendada para intentar evitar que la persona obstruya sus vías respiratorias cuando se encuentra inconsciente, siempre cuidando que no hubiera otra situación que pudiera amenazar su vida en ese momento.

Puede resultar de interés la consulta de un glosario de términos de la OMS para referirse al abuso de sustancias (*https://www.who.int/substance_abuse/terminology/lexicon_alcohol_drugs_spanish.pdf*)
